# ‘Nobody comes to help us’: lived experiences and needs of older adults who lost their only child in China

**DOI:** 10.1080/17482631.2022.2153424

**Published:** 2022-12-05

**Authors:** Ni Ning, Chenyang Peng, Meiling Qi, Xiaoping Li, Mei Sun

**Affiliations:** a Xiangya School of Nursing, Central South University, Changsha, China; b The Second Xiangya Hospital, Central South University, Changsha, China; c Hunan University Library, Hunan University, Changsha, China; d Xiangya Center for Evidence-Based Practice & Health care Innovation, A Joanna Briggs Institute Affiliated Group, Changsha, China

**Keywords:** Only child, bereavement, childless, older adults, life needs, qualitative study, content analysis

## Abstract

**Purpose:**

This qualitative study aimed to gather insights into the experiences of older adults after losing their only child and explore meaningful life needs as a basis for social interventions.

**Methods:**

We conducted individual face-to-face interviews with 29 participants from 10 communities in Changsha, Hunan Province, China. Interviews were transcribed verbatim and then analysed using the inductive category development of conventional content analysis.

**Results:**

The experience of losing an only child was devastating and linked with a helpless life in old age. The analysis generated the following three themes encompassing their lived experiences and needs: afraid of getting sick, lying on the edge of misery and surrounded by loneliness.

**Conclusions:**

Losing an only child triggered older adults’ feelings of being misunderstood, disconnected and hopeless. They had an increased likelihood of lacking more on love and belonging, esteem and self-actualization needs than their physiologic and safety needs. Findings from our study will raise awareness on this vulnerable group and help design intervention programmes targeting the specific needs of this neglected segment of the population.

## Introduction

The Chinese government implemented the one-child family planning policy in 1979 to control the population, adapting it to economic and social development. However, it also brought a new phenomenon of “childless” older adults who had no living birth or adopted child after the only child’s death caused by injuries or illnesses (Li, 2013). This group is expanding rapidly in China. With an annual growth of 76,000, childless families are expected to reach 10 million by 2035 in China (Wang & Hu, 2020). As a special group of the elderly, not only do childless older adults endure severe and long-lasting loneliness, they face many life challenges that threaten their physical, mental and social health, resulting in a higher prevalence of suicidal ideation (SI). The prevalence of SI among Chinese older adults who lost an only child was as high as 11.24% (Wang et al., 2019).

Many researchers identified that losing a child was a devastating experience for parents and associated with considerable functional impairment and adverse health outcomes. A narrative literature review suggested a higher risk of mortality, mental disorders and life stress among bereaved parents, which were reported to be the highest early on after the child’s death and gradually eased with time as parents adjusted to their child’s death (October et al., 2018). A prospective population-based study in Sweden also concluded that parents who experienced offspring death had more than a 10-fold higher risk for mental disorders (Wilcox et al., 2015). However, most western studies in this field only focused on the death of one child, regardless of the total number of children (Wang, Hu, 2021). Compared with western countries, the only-child death problem seems to be more complex and requires discussions in the Chinese unique cultural and political contexts.

Confucian ideology and familism culture, which advocate filial piety and continuity of the family line as a life mission, are both deeply rooted in the Chinese tradition (Lou & Ng, 2012; Zhang & Jia, 2018). Thus, losing an only child equals no progeny, which is stigmatized as extremely unfilial in China (Wang & Hu, 2020). This severe cultural stigma not only leads to increased social discrimination and reduced social support but also causes diminishing self-esteem, which is then associated with a high prevalence of SI (Wang et al., 2019; Zhang & Jia, 2018; Zheng et al., 2017). Inevitably, childless parents become isolated in Chinese society and thus develop profound social loneliness.

Moreover, the one-child policy prompted a special social phenomenon in China, which is child-centredness (Wang et al., 2021). Parents invested a large amount of love, energy, money and time into their only child who held the entire family’s expectations (Xu, 2015). The only child’s death terminated the main source of spiritual and financial support. Eventually, this incomplete family drove couples to divorce, causing the breakdown of family functions (Wang & Hu, 2020; Wang, Chaiyawat, 2021; Zheng et al., 2017). Losing an only child has become a special family risk in China.

As parents grow older, some most likely need assistance from adult children who serve as their primary caregivers. Especially in China, ageing parents live with their children and mostly rely on children’s support (Zheng & Lawson, 2015). Parents who have lost their only child are faced with the lack of potential support. Thus, focusing on the supportive needs of older adults who lost their only child is important. Previous limited quantitative studies showed that most of them had care, medical coverage, emotional and social support needs. Emotional support accounted for the highest proportion of all needs (An, 2014; Wang et al., 2017). Powers (2006) suggested that “need” was a statement made by a person, and needs were subjective. Determining the life needs of older adults who lost their only child is necessary for individual interviews. A comprehensive understanding of their needs after losing their only child can assist healthcare providers, who play a central role in their care to help alleviate grieving parents’ distress (Driscoll, 1990; Liao & Ma, 2014; October et al., 2018) in providing better care for childless older adults. However, to our knowledge, no qualitative studies of childless older adults’ life needs have been published.

Maslow’s hierarchy of human needs serves as a useful framework for analysing data on supportive needs of older adults who lost their only child. He depicted five hierarchies of human needs: physiologic needs, safety, love and belonging, esteem, and self-actualization as the needs that motivate and guide individual behaviour. The needs in the lower hierarchies must be at least partly satisfied before pursuing the higher hierarchies. Maslow believed that every person differs in their pursuit of needs; in every period of a lifetime, a person has a dominant need (Maslow, 1943). Maslow’s theory remains a common application for sociology and psychology (Benson & Dundis, 2003) and offers a framework to determine the needs that are important to older adults who lost their only child.

This study is unique in combining Chinese cultural characteristics, Maslow’s hierarchy of needs and collaboration with a civil society organization named, “Childless Club”, which is needed to succeed with coproduction in the research on vulnerable populations (Kneck et al., 2021). This study aims to gather insights into the experiences of older adults after losing their only child and to explore their meaningful life needs.

## Methods

### Study design

Considering the ultimate goal of this qualitative study, the researchers chose the conventional content analysis as a preferred method to obtain additional knowledge about the experiences of older adults who lost their only child and then determine effective solutions to their needs. Conventional content analysis is used with a study design whose aim is to describe a phenomenon and provide knowledge and understanding of the phenomenon by interpreting meaning from the content of text data (Hsieh & Shannon, 2005). The advantage of the conventional content analysis is gaining direct information from study participants structured to capture the maximum diversity of lived experiences and needs. This report adheres to the Consolidated Criteria for Reporting Qualitative Research (COREQ) (Tong et al., 2007).

### Participant selection

We recruited the participants aged over 60 years old with the experience of losing their only child in Changsha, the capital city of Hunan Province, China. Those with cognitive impairment and serious mental or physical illnesses were excluded. The authors contacted potential participants through a local civil society organization via purposive sampling and snowball sampling. Participants with maximum variations in demographic data, such as gender, age, marriage status and the number of years since they lost their only child were targeted. The life expectancy of the Chinese is gradually increasing, with 36.6 million Chinese aged 80 and over in 2020 (Ren, 2022). We included the participants aged over 80 to achieve maximum variation in age. Potential participants were informed about the study via telephone, including our interest to interview older adults who lost their only child, their perspectives on life needs and their right to withdraw from the study at any time. Researchers communicated with the head of the organization to ensure that all suitable parents were provided with an opportunity to participate. During the recruitment period, the researchers identified 33 people who met all inclusion criteria. Twenty-nine people agreed to join the study. Reasons for not participating included the lack of time (n = 2) and health issues (n = 2).

### Data collection

Face-to-face, in-depth, individual interviews lasting for approximately 40–58 mins were conducted by NN, CYP and MLQ, all of whom were trained female psychological researchers and a social worker assistant in participants’ homes between Jan 7 and 20 February 2020. Each participant was interviewed once, and some of them were couples who were simultaneously interviewed once independently in separate rooms.

NN and CYP designed an interview outline, which was then refined and sorted out by MS who was a senior qualitative study researcher. The interviews were performed in Mandarin and were audio recorded. Before the formal interviews, pre-interviews were conducted twice to modify the outline. We changed the question, “Can you handle most of the difficulties in life by yourself?” to “How did you overcome the difficulties?” We also deleted the question. “Can you tell us how you felt when your child left?” after the pre-interviews. The final main questions asked in the interviews were as follows: What difficulties have you encountered in your life? How did you overcome the difficulties? Have you received any help at difficult times? What is the most effective way to help you? Probing questions were also asked to further scrutinize childless older adults’ ideas and clarify their responses during the interviews, such as follows: Would you explain more about this? What is the meaning of that notion? Could you please give an example to help us correctly understand your point?

Apart from the audio record, researchers (NN, CYP, and MLQ) also took field notes to keep a record of the participants’ facial expressions, body language and important responses during the interviews.

The data collection and analysis proceeded concurrently. After interviewing 27 participants, data saturation was considered to have been attained. Two additional participants were interviewed to confirm that no new themes have emerged.

### Data analysis and rigor

NN, CYP and MLQ transcribed verbatim all the audio records within 24 hours after the interviews to reduce the risk of selective data filtering through recall. In addition, the transaction draft and field notes were analysed in NVivo11 using conventional content analysis as described by Elo and Kyngäs (2008) and Hsieh and Shannon (2005).

The first authors (NN and CYP) read and re-read all de-identified transcriptions of the interviews (n = 29) and divided all texts into meaning units. Considering the context, the meaning units were condensed into a description close to the text. The manifest content was interpreted into the underlying meaning, that is, the latent content. The condensed meaning units were seen as a whole and abstracted into sub-themes. The latter was further combined into three themes. Inconsistent opinions were resolved by inviting MS into a discussion until a consensus was reached. Social workers also reviewed the entire transcripts of assigned (n = 2) and randomly chosen (n = 4) interviews as well as the sections of most interviews. It was an iterative process in which the data were reused multiple times until the researchers and the society organization reached a common understanding. The themes were presented to MS, MLQ and Dr. Wang who studied on the mental health of childless parents and were then revised with consideration of their opinions (See Tables 1 for examples of analysis proceedings).

**Table 1.** Analysis process examples.

Meaning unit	Condensed meaning unit	Interpretation	Sub-theme	Theme
*I’m afraid of measuring my blood sugar and of being told my blood pressure is high … . (about physical examination) I won’t go to the hospital. I don’t want to deal with my health problems, just leave them alone … , I don’t mean I’m frightened to die, but I’m afraid of nobody taking care of me … , you know we have nobody to rely on. It’s helpless …*	I’m afraid of having physical examination and dealing with my health problems. I’m not afraid of death but of having nobody to rely on. It’s helpless for us.	The reason why I’m afraid of getting sick is I lost my only child and have nobody to rely on. It’s helpless if I get sick.	Nobody to rely on	Afraid of getting sick
*My brother told me that my mother was in danger when I was in the hospital. I immediately left the hospital without discharging formalities to take care of my mother…My mother passed away after four months. I told my father that I didn’t feel well; so, I went to the hospital after being unwell for six months … My father is in poor health now; nobody takes care of him except me.*	Even though I didn’t feel well, I immediately left the hospital to take care of my mother who was in danger. I went to the hospital until my mother passed away. My father is in poor health now. However, nobody takes care of him except me.	I don’t have time to have my disease treated. If I am in the hospital, nobody can take care of my parents.	Responsibility to take care of the older parents	
*I don’t want to take any physical examination. Once examined, it will certainly reveal some problems and cost me time and money … I am afraid of never being able to look after my grandchild, he is too young. If I get sick, who can raise him (frowning) …*	Physical examination will reveal my health problems which cost time and money. If I get sick, who can raise my little grandchild?	What’s scary is not that I’m sick but that nobody can raise my grandchild.	Responsibility to raise the third generation	

Several strategies were used to ensure trustworthiness and credibility (Birt et al., 2016; Lincoln & Guba, 1985). Two authors (NN and CYP) independently analysed the results to recognize the initial codes before comparing their codes and categories. The credibility of data was further established through member check. We prepared a concise report on emerging themes along with interview data quotes which presented the themes returned to the participants. Before sending back the member checking documents to the participants, we checked with the social worker whether the participants were in good health to reduce the risk of distress. Participants were asked to read the document and check whether they felt that the composite results resonated with their experience. All participants agreed with the member check, and no changes were made to the analyses.

The researchers wrote reflective diaries after every interview to achieve the reflexivity of this study. The researchers learned that they eased the participants’ nervousness as the collaborating social organization familiar to the participants. Every researcher discussed their reflective diary with qualitative expert consultants. The reflective diary also contributed to maintaining and guiding the decision-making process about the study.

### Ethical considerations

All participants were offered written and verbal information about the study, and written informed consent was provided by all participants. No identifying personal information that could be connected to a specific childless older adult was collected. All data were self-reported, and no medical files were accessed. Audio recordings and all written documentations of research data were stored in a password-protected folder only accessible to the research group. Ethical approval was granted by the IRB of behavioural and nursing research (2018041).

## Findings

Our samples consisted of 19 females and 10 males. The participants’ ages ranged from 60 to 81 years, and the time since their child’s death ranged from 1 to 10 years. More than half (n = 23) were married and included five couples. Only eight participants had no diagnosed disease, and the remaining participants (n = 21) all had diagnosed diseases (see Table 2).

**Table 2.** Social-demographic characteristics of the participants.

Participant	Gender	Age	Marriage status	The number of years since the child’s death	Diagnosed disease
N1	female	60	without a spouse	2	Arteriosclerosis, diabetes
N2	male	62	with a spouse	3	Hypertension, diabetes, heart failure
N3	female	61	without a spouse	1	Thyroid nodules
N4	femal	64	with a spouse	4	Diabetes, lupus erythematous, fatty liver
N5	female	81	without a spouse	5	Diabetes
N6	female	62	with a spouse	3	Arrhythmia
N7	female	60	with a spouse	2	Anaemia, diabetes
N8	female	61	with a spouse	1	Metro carcinoma
*N9	male	62	with a spouse	3	
N10	male	72	with a spouse	4	
N11	male	60	with a spouse	5	Coronary heart disease, diabetes, hyperglycaemia
N12	male	69	with a spouse	4	Hypertension, gout, fatty liver
N13	female	61	without a spouse	3	Chronic pharyngitis, urinary incontinence
*N14	male	64	with a spouse	1	
*N15	female	63	with a spouse	1	
*N16	female	65	with a spouse	2	Thyroid nodules
*N17	female	62	with a spouse	3	Bronchitis
*N18	male	64	with a spouse	3	Hypertension
*N19	female	69	with a spouse	2	Nerve deafness
*N20	male	71	with a spouse	2	Hypertension, diabetes, hyperlipidaemia
N21	female	60	with a spouse	1	
*N22	female	67	with a spouse	2	Diabetes
*N23	male	69	with a spouse	2	
N24	female	64	with a spouse	3	Coronary heart disease
N25	female	69	with a spouse	1	Thyroid nodules, hypertension
N26	female	71	without a spouse	1	Thrombocytopenia, hypertension
N27	female	66	with a spouse	1	
N28	male	63	without a spouse	10	
N29	female	62	with a spouse	1	Superficial gastritis

*Note: N9 and N16, N14 and N15, N17 and N18, N19 and N20, N22 and N23 were couples.

**Figure 1.**
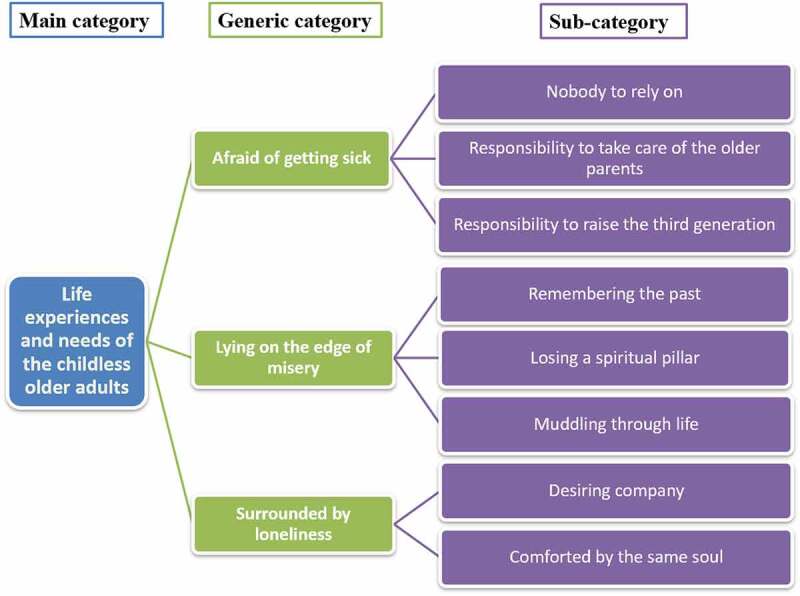
Abstraction processes.

Three themes were identified through analysis: afraid of getting sick, lying on the edge of misery and surrounded by loneliness (see Figure 1). Overall, the parents described experiencing extreme pain after losing their only child but also found a way to cope with it overtime. The next section thoroughly discusses the three themes and their subthemes with illustrative quotes from the participants identified in italics.

### Theme 1: Afraid of getting sick

The theme highlights that the participants have a desire to stay healthy, which is related to the following three subthemes: (a) nobody to rely on, (b) responsibility to take care of the older parents and (c) responsibility to raise the third generation.

#### Subtheme (a): Nobody to rely on

An old saying in China states that raising children is a guard against one’s old age. Old parents had no one to rely on after losing their only child. The interviewers stated that they were not afraid of death but of not being cared for as they age and progressively deteriorate. Dealing with the pressure and the burden of disease alone was difficult for them without the younger generation’s assistance, which triggered their fear of facing their actual health status:

*I don’t want to deal with my health problem … I don’t mean I’m frightened to die, but I’m afraid of nobody taking care of me … You know we have nobody to rely on. It’s helpless … (Crying) (N7)*

*It is too complicated and hard for me to see a doctor. I dare not get sick. I wish that our government can send someone to take care of the sick elderly like us. However, there is no policy to implement and any other people come to help us …* (N25)

#### Subtheme (b): Responsibility to take care of the older parents

In a traditional Chinese family setting, filial piety is rigidly observed. Adult children have the responsibility to care for their older parents. Without the younger generation’s help, those childless parents have to embrace all the responsibilities to care for their older parents by themselves, even if they are sick. Accordingly, they hoped to stay healthy for fear of not being able to fulfill such responsibilities. A widow revealed that she had to abruptly end her treatment to care for her parents because her brother lived far away and was too busy to assume such a caregiving role.

*Life for me is a continual struggle … I don’t have time to take care of myself … My brother told me that my mother was in danger when I was in the hospital. I immediately left the hospital without discharging formalities to take care of my mother… My mother passed away after four months … My father is in poor health now; nobody takes care of him except me*. (N13)

#### Subtheme (c): Responsibility to raise the third generation

Some of the interviewees’ children including their children’s spouses died from accidents, consequently leaving the interviewees’ grandchildren orphaned. Traditional Chinese culture advocates people to treat the continuation of the family line as a life mission. For these childless old adults, the grandchildren were the only chance of family blood inheritance. Therefore, under the influence of traditional Chinese culture, they felt compelled to do their best in raising the grandchildren after losing their only child. However, the interviewees had to raise the younger grandchildren whilst caring for their elderly parents, which not only forced them to ignore their own health conditions but also urged them to make a living for their families.

*I don’t want to take any physical examination. Once examined, it will certainly reveal some problems … I am afraid of never being able to look after my grandchild; he is the only hope of our family. If I get sick, who can raise him (frowning)?* (N11)

### Theme 2: Lying on the edge of misery

Nearly all the interviewees described their painful experience of losing their only child. Three subthemes were extracted from the conversations, including (a) remembering the past, (b) losing a spiritual pillar and (c) muddling through life.

#### Subtheme (a): Remembering the past

Most of the participants mentioned that when they saw something associated with their child, they remembered those days when their only child was still alive. They were immersed in their past worlds and felt inexorably trapped in a swamp of grief. They attempted to escape from those memories but failed:

*It seems that there is a film in my mind, wherever I am. Whatever I think, I can’t get around those things (lost the only child). Once, I hung around and saw other people’s young children … memories of my son filled my mind … I try my best to not think about that, but I can’t …* (N13)

*I need a new environment. If I stay with others’ children, I may feel … been reminded … I sold my house and moved to another place after my son passed away because I felt uncomfortable in the old one*. (N24)

#### Subtheme (b): Losing a spiritual pillar

In China, parents hold high expectations for their children and regard their only child as a spiritual pillar. Parents felt they lost emotional support, and recovering from the trauma took a long time after losing their only child. Some of the interviewees who had lost their only child more recently failed to cope with the misfortunes and even experienced a mental breakdown. They have not yet found their new inner resource. Seven interviewees indicated that their lives became dull and meaningless after losing their only child. Meanwhile, the participants who lost their only child for a long time have found activities that interest them to fill the emptiness.

*I used to dance, but I never go dancing after that happened (lost an only child). I had no desire to dance. I forget my dance movement sometimes*. (N27, lost an only child 1 year ago)

*I’m always in touch with my friends. I played cards with my friends yesterday; they (volunteers) visit me a lot, and they always call me “Happy Grandma.”* (N5, lost an only child 5 years ago)

#### Subtheme (c): Muddling through life

Losing an only child can be devastating. Following their only child’s death, parents became utterly overwhelmed, discouraged and self-paralysed. Older adults who lost their only child demonstrated different ways of escaping reality, and these coping methods were particularly distinct between male and female: five women went through months of crying, and one of them thought that belief in Buddhism made her life fulfilling. Meanwhile, all of the male interviewees comforted themselves by smoking or drinking alcohol.

*I have got a headache because of crying every day after my daughter’s death*. (N25, female)

*I practice Buddhism every day and night since my daughter got sick. I’m not a religious believer before that … Thanks to Buddhism, I must be crazy if I don’t practice it*. (N15, female)

*I just can’t control myself. I drank alcohol at least 500 mL per day. You know, because of my family, I numbed myself with alcohol everyday …* (N2, male)

*I know smoking is bad for my health, but I can’t control myself. I smoke cigarettes when I feel annoyed*. (N10, male)

### Theme 3: Surrounded by loneliness

This theme describes the loneliness of childless older adults and illustrates how they coped with it. These experiences are linked to two subthemes including (a) desiring company and (b) comforted by the same soul.

#### Subtheme (a): Desiring company

Nearly all participants expressed their desire for companionship. Couples who lost their only child supported each other through difficult times. They believed that they had experienced the grief together and were the ones who understood each other best. Participants without a spouse felt a stronger sense of loneliness and had a more intense longing for companionship. They had to come out of the spiritual world of self-isolation and seek out like-minded partners.

*My husband can understand my pain, and we often talk about why the demise of our son happened … we comfort each other when we miss our son, at least we have each other’scompany …* (N4, with a spouse)

*I felt empty at home alone. I want to do things of interest with other friends who understood me … I am very happy that volunteers chat with me. I look forward to their visits every time*. (N13, without a spouse)

#### Subtheme (b): Comforted by the same soul

Some participants commented that they preferred to make friends with someone who had the same experiences. They believed that friends who experienced the same difficult time can understand their pain and can better help alleviate their suffering. The Childless Club helps older adults who lost their only child establish friendships with people who had the same experiences by providing a platform for them. Moreover, the interviewer found that older adults who had lost their only child for a long time can support the ones who did so more recently to relieve the grief and adjust to the loss of a loved one:

*The first time I joined the organization, they said we are the same person. All of them are strong-minded, which encourages me a lot*. (N8, lost an only child 1 year ago)

*I participated in nearly all the activities in the organization (Childless Club). I enlightened them when they were helpless or sad*. (N12, lost an only child 4 years ago)

*I know how dark their daily lives are because I’m the one who overcame a similar fate. It’s my duty to help them overcome these hard days and nights*. (N29, lost an only child 10 years ago)

## Discussion

Older adults who lost their only child were victims of the one-child policy and were deeply affected by the loss of their beloved. Interview findings attested older adults who lost their only child had these five hierarchical needs: physiologic needs, safety, love and belonging, esteem and self-actualization. In addition, they were more likely to lack higher-level needs than their physiologic and safety needs under a Chinese well-developed social security system, which is consistent with Maslow’s hierarchy of human needs theory.

Forty-seven Chinese policies related to parents who lost their only child have been issued since 2001. A nationwide policy providing monthly subsidies, ranging from 200 RMB (30 $) to 800 RMB (120 $), is the most popular one (Wang et al., 2017). In Changsha, where the data have been collected, childless older adults receive 400 RMB (60 $) per month. However, these policies do not cover the mental health area and therefore cannot fully meet the childless older adults’ actual needs (Xiong et al., 2021). Accordingly, we suggest that healthcare providers implement the targeted strategies according to the experience of childless older adults, which is a crucial measure to achieve the sustainable development of ageing societies. Consequently, the forthcoming discussion aims to direct healthcare providers towards this end on the basis of the five hierarchical needs depicted by Maslow.

### Physiologic and safety needs

Our findings are consistent with previous studies that older adults who lost their only child had considerable physical and psychological impairment (Yin et al., 2018). However, our findings suggest that some of them had a strong need to stay healthy and seek easier access to medical resources, which were somewhat different from Li, 2013’s study that childless older adults had lost their desire to live and were even suicidal. We consider the following reasons to explain this phenomenon in our study. First, Li’s study conducted in Beijing, the capital of China, had higher living expenses than Changsha where our interview data were collected. Childless older adults in Beijing may have difficulty in maintaining survival due to huge financial pressures after losing the support of their only child. Second, Li’s study was conducted five years ago when researchers and policy makers did not take any measures to support childless older adults. Finally, the traditional Chinese culture advocates the continuity of the bloodline. The old motto “there are three forms of unfilial conduct, of which the worst is to have no descendants” is preserved by Chinese society from generation to generation (Li, 2013; Zhang & Goza, 2006). Older adults who are still living with the third generation emerge with a strong sense of mission to care for the latter and thus continue the family bloodline after losing their only child. However, they encountered many difficulties in maintaining good health due to their age and lack of knowledge.

A number of studies noted the effectiveness of primary care in bereavement care (Li, 2005; Wang et al., 2017). However, primary health management of older adults in China is based on the disease classification, lacking attention and protection for childless older adults. Accordingly, we suggest that primary healthcare providers first establish personal health records to grasp childless older adults’ physical and psychological dynamic changes, a practice very common in Singapore and Japan (Kang, 2017). Regular visits to childless older adults and providing targeted health education for someone with diseases are necessary to raise their awareness on how to avoid worsening health conditions and when to seek medical attention. Social security and medical insurance reimbursement rates should also be improved for them, given that most of them pay higher medical expenditures due to severe physical diseases (Xiong et al., 2021). For someone who has mental health problems, implementing professional psychological intervention, employing cognitive strategies to re-frame negative feelings and providing free psychological counselling and regular psychiatric screening are necessary (Kharicha et al., 2021).

### Love and belonging needs

Another unexpected finding is the paradox of self-isolation and the longing for companionship. Most studies suggested that parents who lost their only child withdrew from friends and relatives, feeling that others failed to understand them and confining themselves to a limited social network (Li, 2013; Wang, Chaiyawat, 2021; Zhang et al., 2019). Our study found that although childless older adults isolated themselves, they were willing to associate with like-minded friends or relatives and chat with social workers, which is consistent with the research of Meert et al. (2009) and Shattell and Hogan (2005). This finding may be related to the active presence of hope in vulnerable populations, regardless of their dire circumstances which can be described as seeking understanding in isolation and helplessness (Partis, 2003; Zhang et al., 2019). In our study, we found that marriage might improve individual negative emotion after losing an only child, which is consistent with a cross-sectional study also conducted in Changsha (Zhang et al., 2016). Older adults without a spouse had a stronger need for others’ companionship and understanding than those with a spouse. A committed partner who shared the same experience in a stable family can offer beneficial support to increase the sense of love and belonging. However, a former survey conducted in 10 areas of China found that married women who have lost their only child were more likely to suffer from depression than unmarried ones (Wang, Chaiyawat, 2021). Further research is needed to explore marriage in moderating the negative emotion among couples who lost their only child.

The tremendous pressure of losing the only child has created new social interactions and emotional needs (Shattell & Hogan, 2005). As a non-government society organization, the Childless Club played a huge role in meeting the love and belonging needs of older adults who lost their only child (Li, 2013; Xiong et al., 2021). They worked with volunteer teams to provide childless older adults door-to-door service. More importantly, they constructed a platform for those older adults with the same experience to engage in the activities that interest them and deal with their grief through peer support. Therefore, healthcare providers should perceive and use childless older adults’ internal hope as a resource to effectively protect them from social isolation (Zhang et al., 2019) and improve cooperation with non-government society organization in organizing group outings for older adults who share the same experience of losing an only child to encourage seeking other joys in life to alleviate their loneliness (Morlett Paredes et al., 2021; Zhang et al., 2017).

### Esteem and self-actualization needs

Our study identified that childless older adults welcomed the volunteers’ house-call visits and hoped that volunteers chat with them more frequently, especially for those who were not good at making friends. Unlike others who discriminate against them, volunteers were willing to listen to what they said. They wanted others to respect and recognize them (Zheng & Lawson, 2015). In addition, childless older adults prefer to share their concerns and anxieties to the permanent volunteers who were familiar with them. At the same time, older adults who lost their only child for a long time can help those who recently lost an only child adjust to the loss of a loved one, which granted them self-satisfaction as they perceived to have actualized their rightful value and found the meaning of living. The outcome of Dong and Chen (2014)’s study was consistent with the current research finding that some childless people were willing to join support groups to support others who had the same experience by playing the role of rights defenders and organizers. A number of studies also suggested that peer support was an effective intervention for bereaved people (Barlow et al., 2010; Dopp & Cain, 2012; Rudd & D’Andrea, 2013).

Therefore, such individuals who provide peer support should be encouraged to be included in support programmes. Whilst pursuing self-actualization, they are also helping their peers escape from the depth of misery. Moreover, recruiting fixed volunteers who will maintain long-term contact with older adults who lost their only child is beneficial to build a trusting relationship with each other (Li, 2021). However, uniting the power of volunteer teams is a challenge because the public’s understanding and attention to older adults who lost their only child remain limited (Yao et al., 2016). A society organization that closely stays in touch with these older adults can serve as “advocates”, connecting the volunteer teams to provide extra attention and assistance for childless older adults by writing summary reports, making short videos and taking photographs through owned media.

### Limitation

Limitations of this study include the following two aspects. Firstly, only childless older adults were interviewed, and the experiences of social workers and volunteers were excluded. Further interview efforts can be made to examine the perspectives of other social workers and volunteers who connected with the childless older adults, which may provide a more comprehensive and deeper interpretation on how to support childless older adults. Secondly, this study relies on the data from Changsha childless older adults with middle average financial subsidies that may have higher homogeneity. Interview samples from different cities in China would be interesting.

## Conclusions

In general, older adults who lost their only child live at the outer edge of society. The experience of losing their only child triggers their feelings of being misunderstood, disconnected and hopeless. They have multiple specific supportive needs, especially for love and belonging, esteem and self-actualization needs. We suggest that healthcare providers increase their cooperation with non-government society organizations, lead the way by speaking up to attract the public’s attention and restore the happiness of this population in caring for and establishing mutual understanding relationships. Findings from our study will help design intervention programmes that will target the specific needs of this neglected segment of the population. This effort is imperative to ease the estrangement and indifference between the older adults who lost an only child and others, avoid social conflicts and promote the sustainable development of our societies.
